# Factors influencing integration of mental health screening and treatment at HIV clinic settings in Cameroon: a qualitative study of health providers’ perspectives

**DOI:** 10.1186/s12913-024-10775-w

**Published:** 2024-04-24

**Authors:** Kathryn E. L. Grimes, Peter Vanes Ebasone, Anastase Dzudie, Denis Nash, Milton L Wainberg, Brian W. Pence, Clare Barrington, Eric Pefura, Marcel Yotebieng, Kathryn Anastos, Denis Nsame, Rogers Ajeh, Annereke Nyenti, Angela M. Parcesepe

**Affiliations:** 1 Department of Health Policy and Management, Gillings School of Global Public Health, University of North Carolina at Chapel Hill, Chapel Hill, NC USA; 2 Clinical Research Education Networking and Consultancy, Yaoundé, Cameroon; 3 Institute for Implementation Science in Population Health, City University of New York, New York, NY USA; 4 Department of Psychiatry, Columbia University Vagelos College of Physicians and Surgeons, and New York State Psychiatric Institute, New York, NY USA; 5 Department of Epidemiology, Gillings School of Global Public Health, University of North Carolina at Chapel Hill, Chapel Hill, NC USA; 6 Department of Health Behavior, Gillings School of Global Public Health, University of North Carolina at Chapel Hill, Chapel Hill, NC USA; 7 Jamot Hospital, Yaoundé, Cameroon; 8 Department of Medicine, Albert Einstein College of Medicine, Bronx, NY USA; 9 Departments of Medicine and Epidemiology & Population Health, Albert Einstein College of Medicine, Bronx, NY USA; 10 Bamenda Regional Hospital, Bamenda, Cameroon; 11 Limbe Regional Hospital, Limbe, Cameroon; 12 Department of Maternal and Child Health, Gillings School of Global Public Health, University of North Carolina at Chapel Hill, Chapel Hill, NC USA; 13 Carolina Population Center, University of North Carolina at Chapel Hill, Chapel Hill, NC USA

**Keywords:** HIV, Mental health, Health providers, Service integration, Cameroon, Qualitative research

## Abstract

**Background:**

Mental disorders are common among people with HIV (PWH) and are associated with poor HIV outcomes. Despite high unmet mental health needs among PWH, use of evidence-based mental health screening and treatment protocols remains limited at HIV treatment facilities across low-resource settings. Integrating mental health services into HIV care can reduce this gap. This study’s objective was to explore factors that influence integration of mental health screening and treatment into HIV clinics in Cameroon.

**Methods:**

We analyzed 14 in-depth interviews with clinic staff supporting PWH at three urban HIV treatment clinics in Cameroon. Interviews focused on current processes, barriers and facilitators, and types of support needed to integrate mental health care into HIV care. Interviews were recorded and transcribed. French transcripts were translated into English. We used thematic analysis to identify factors that influence integration of mental health screening and treatment into HIV care in these settings. Ethical review boards in the United States and Cameroon approved this study.

**Results:**

Respondents discussed a lack of standardized mental health screening processes in HIV treatment facilities and generally felt ill-equipped to conduct mental health screening. Low community awareness about mental disorders, mental health-related stigma, limited physical space, and high clinic volume affected providers’ ability to screen clients for mental disorders. Providers indicated that better coordination and communication were needed to support client referral to mental health care. Despite these barriers, providers were motivated to screen clients for mental disorders and believed that mental health service provision could improve quality of HIV care and treatment outcomes. All providers interviewed said they would feel more confident screening for mental disorders with additional training and resources. Providers recommended community sensitization, training or hiring additional staff, improved coordination to manage referrals, and leadership buy-in at multiple levels of the health system to support sustainable integration of mental health screening and treatment into HIV clinics in Cameroon.

**Conclusions:**

Providers reported enthusiasm to integrate mental health services into HIV care but need more support and training to do so in an effective and sustainable manner.

**Supplementary Information:**

The online version contains supplementary material available at 10.1186/s12913-024-10775-w.

## Background

Mental disorders, such as depression, anxiety, post-traumatic stress disorder (PTSD), and substance use disorders are common among people with HIV (PWH). Studies have found that PWH are more likely to experience mental disorders compared to the general population [1, 2]. A systematic review and meta-analysis found the pooled prevalence of depression symptoms, the most common mental disorder among PWH, ranged between 14 and 32% among PWH on antiretroviral therapy (ART) in sub-Saharan Africa (SSA) [2]. A separate systematic review and meta-analysis estimated the prevalence of anxiety to be between 3 and 70% among PWH in low- and middle income countries (LMICs) [3]. In addition, psychiatric multimorbidity, or the presence of two or more co-occurring mental disorders, is common among PWH [4, 5]. Research suggests the high prevalence of mental disorders among PWH may be influenced by HIV-related stigma and worry about the impact one’s HIV status has on interpersonal relationships and economic stability [6]. Mental disorders have been associated with poor HIV treatment outcomes, including delayed HIV treatment initiation, suboptimal adherence to ART, and poor retention in care [1, 3, 7–11]. Despite the high prevalence of mental disorders among PWH and the relationship with suboptimal HIV treatment outcomes, integration of mental health care into HIV care remains limited throughout SSA [12, 13].

There is extensive unmet need for mental health care globally, especially in resource-limited settings. A 2017 study in 17 countries found that mental health service use was lowest in low- and middle-income countries (e.g., 1.6% among respondents in Nigeria) through use remained low in high income countries as well (e.g., 17.9% among respondents in the United States) [14]. Health system constraints that impact the availability of mental health screening and treatment include resource scarcity, limited workforce capacity, inefficiency, and inequity [15]. Inadequate human resources is a primary challenge in many low-income countries where there is an estimated median of only 0.05 psychiatrists per 100,000 population [15]. To improve access to mental health care globally, leaders and experts have recommended addressing human resource limitations (including capacity-building and task shifting to non-specialists), decentralizing mental health services, greater investment in mental health infrastructure, and improved coordination across different levels of the health system [16, 17]. While these recommendations have shaped national mental health policies globally, implementing these strategies has proven challenging [17].

Integrating mental health services into HIV care in resource-constrained settings is a potential avenue to support PWH with mental disorders and reduce the mental health treatment gap [18, 19]. By integrating and coordinating mental health care, providers may more consistently identify clients with mental health symptoms, clients’ economic hardship may be alleviated through fewer appointments and reduced transportation costs, and health system navigation may be simplified [18]. In practice, integrating mental health into HIV services remains limited. A survey of 228 HIV treatment sites across 41 countries found that combined screening and on-site counseling was available for depression, anxiety, and PTSD at 50%, 14%, 12% of facilities, respectively [20]. More research is needed to identify barriers and facilitators to effective mental health and HIV service integration.

Expanded services to support PWH in Cameroon with mental disorders are urgently needed. HIV prevalence among adults ages 15–49 in Cameroon is 2.9% [21], though prevalence varies by region [22]. Among PWH initiating HIV care in Cameroon, moderate to severe symptoms of depression, anxiety, and PTSD were reported by 20%, 12%, and 15% of PWH, respectively [10, 11]. Cameroon’s scarcity of mental health professionals and limited resources highlights the pressing need to identify feasible strategies to address mental health needs in the population. The 2016–2027 National Health Sector Strategy reported just two hospitals capable of managing mental disorders and 10 psychiatrists or other mental health professionals in the country [23]. Moreover, recent studies in Cameroon’s West and South West regions revealed stark deficiencies in the availability of mental health screening or treatment protocols: in the Fako Division, only 1.8% of primary health care providers reported they were familiar with a screening tool for depression and 12% reported they regularly screened their clients for depression [24, 25]. Further, while the overwhelming majority (93%) of primary care providers surveyed in Cameroon recognized depression as a condition appropriate for medical care, most (66%) did not feel comfortable treating patients with depression, and more than half (51%) had not received mental health training [25]. Like many resource-constrained settings, the mental health treatment gap in Cameroon warrants increased attention.

Understanding barriers and facilitators to integrating mental health screening and treatment in HIV treatment centers can inform policy and implementation strategies to effectively address the mental health needs of PWH. It is crucial to understand health providers’ perspectives on mental health integration, as they are the key implementers of such strategies. Qualitative research can provide rich context on health providers’ perspectives to ensure their perspectives and needs are integrated into future planning, research, and service delivery and foster successful service integration. The objective of this paper is to explore HIV health providers’ perspectives on factors that influence integration of mental health screening and treatment into HIV care in Cameroon.

## Methods

Qualitative data were collected from health providers in three public urban HIV treatment facilities in Cameroon. These facilities were selected due to their participation in the International epidemiology Databases to Evaluate AIDS (IeDEA) Consortium [26]. Qualitative methods were used as this study was exploratory in nature and sought to obtain preliminary data on a topic about which little was known in this setting. The Institutional Review Board at University of North Carolina at Chapel Hill in the United States and the National Ethical Committee of Research for Human Health in Yaoundé, Cameroon provided ethical approval for this study. Research assistants received training in strategies to maintain participant confidentiality and privacy and obtain informed consent. All participants provided written informed consent. Interviews were conducted in private settings at the study sites.

### Participant selection and sampling

Fifteen in-depth interviews were conducted with health providers across the three study sites. Due to poor audio quality of one interview, data from 14 interviews were analyzed. Participants were eligible for inclusion into this study if they were: (1) providers at study sites who worked with PWH and (2) had been employed at the HIV treatment facility for at least six months. We used purposive criterion sampling [27] to identify providers at each site for interviews with a target sample of five providers at each facility across diverse staffing roles (e.g., clinician, nurse, psychosocial worker). Cameroonian study staff deemed five providers per site a feasible and reasonable sample size given the limited staffing environments at these facilities. This is aligned with Hennink & Kaiser’s (2021) finding that 9–17 interviews are generally sufficient for reaching saturation [28]. As the current study had a homogenous study population and narrowly defined study objective, the research team determined a priori that 15 participants was sufficient for reaching saturation.

### Data collection

Cameroonian study staff trained in qualitative data collection conducted interviews with providers in French or English in a private setting at the facility using a semi-structured interview guide. Interviews occurred between October and December 2021. Each interview lasted approximately one hour. Interviewers asked open-ended questions about current processes to screen for mental disorders and barriers to and types of support needed to screen clients for mental disorders (Additional file 1). The interview guide was developed by a team of US and Cameroonian researchers, health care providers, and mental health experts and was informed by existing literature regarding barriers and facilitators to integration of mental health screening and treatment into primary and HIV health care settings. Interviews were audio recorded.

### Data analysis

We employed a multi-step process to transcribe audio and check for accuracy. For interviews conducted in French, a native French speaker first transcribed interviews and then translated the transcription into English. US-based study staff (KG), a non-native French speaker with professional French proficiency, compared French transcripts to the audio recordings and reviewed English translations for accuracy. PVE, a Cameroonian physician and research manager for IeDEA Cameroon, reviewed and responded to all translation inquiries. A native English speaker transcribed interviews conducted in English, and KG compared all transcripts to the audio files for accuracy.

We conducted thematic analysis following the process outlined by Braun & Clarke [29]. Our process to develop a reliable coding scheme was informed by the steps outlined in Campbell et al. to establish intercoder agreement [30]. First, KG familiarized herself with the data during the transcription process and through multiple active readings of finalized transcripts. Through notetaking and memo-writing, KG generated a list of inductive codes, definitions, and sample excerpts and developed an initial draft codebook. KG and AP met to discuss the coding scheme and clarified the codes and definitions. In an in-depth round of negotiated agreement, KG and AP double coded a randomly selected subset of transcripts (*n* = 2), compared how codes were applied, identified discrepancies, and reached consensus on how to apply codes in all transcripts. KG independently coded all transcripts in Dedoose [31]. Throughout the coding process, KG and AP met weekly to discuss emerging themes and review code definitions and applications. The final codebook collated codes into potential themes and was used to code all relevant data from the transcripts. Transcripts coded prior to finalizing the codebook were revisited to ensure all codes from the final codebook were consistently applied across transcripts. In addition, KG developed a participant matrix to identify how frequently themes and codes occurred across providers and clinics. Naming and defining themes was an iterative process refined by leveraging theme frequencies from the participant matrix, ongoing memo-writing, and team discussion.

Chaudoir et al.’s (2013) [32] implementation science framework informed the development, implementation, and analysis of this research. This multi-level framework asserts that factors at the structural, organizational, patient, provider, and innovation levels affect successful implementation of evidence-based health innovations and implementation outcomes. In the codebook development phase, we sought to map inductively-identified codes to each level in Chaudoir’s framework. However, several codes did not fit exclusively into one level of the framework. As a result, and motivated by a desire to remain as close to the data as possible, we organized our results by key themes most salient in the data, with consideration of the number of excerpts coded, the depth of the responses, and number of participants endorsing each theme.

KG engaged in ongoing, reflexive memo-writing [33] throughout the transcription, coding, analysis, and writing phases. KG and AP discussed key themes with Cameroonian colleagues to ensure findings resonated with their experiences as physicians and researchers supporting mental health among PWH in Cameroon.

## Results

Results are presented below by key themes identified inductively from participant responses during interviews. The first sections are focused on challenges reported by providers including: limited standard mental health screening processes or tools; mental health-related stigma and limited awareness about mental disorders; physical space constraints and high clinic volume; and the need for better coordination and communication to manage referrals. The final section is focused on provider-reported motivation and confidence to screen for mental disorders.

### Mental health screening processes and tools

When asked about the current process to screen PWH for mental disorders at their facilities, most indicated there was no standardized screening process. One provider said, *“for this number of years that I’ve been working in this treatment center, they have not been screening for mental health” (Provider 1, Clinic 1)*. Providers across all facilities described the ways they worked to address their clients’ mental health needs through informal assessments or based on the client’s appearance or demeanor. Providers described their informal method of screening as: *“[you] see the patients’ reactions, behavior, and their way of thinking” (Provider 4, Clinic 2), “you see them that they are stressed” (Provider 3, Clinic 1)*, and *“when you receive a client, looking at his physical countenance you should be able to identify if he has a problem or not” (Provider 2, Clinic 3).*

Almost all providers (13/14) mentioned the lack of tools and protocols as major barriers to integrating evidence-based mental health screening. Providers shared, *“for mental health in this facility, I’ve not seen the mental health tools or the tools for screening” (Provider 4, Clinic 1).* Without access to evidence-based tools or protocols, providers felt ill-equipped to more formally screen clients for mental disorders. One provider acknowledged limitations in their current mental health screening process:

*“I’m already doing the screening [of] my clients though I’m very certain that I’m not respecting the appropriate protocols because I do not know them. But I’m already doing the screening based on what I said earlier, physical outlook and their history” (Provider 2, Clinic 3)*.

This provider continued to describe how the lack of screening protocols resulted in inconsistent screening.

### Mental health-related stigma and community awareness

Mental health-related stigma was described as a barrier to mental health screening. Providers reported that PWH had “*this fear of being labeled as mad that is also a bit of an obstacle to treatment” (Provider 5, Clinic 2)*. Providers shared that there was stigma associated with specialized mental health facilities and described clients attending these facilities as *“agitated”* because they were afraid someone might recognize them. At one facility, providers mentioned that clients often avoided the mental health unit. Similarly, some providers expressed that they did not want to partner with the mental health unit for fear that others would assume they were mentally ill. One provider described that by just working with clients with mental health problems, *“you [are] ashamed that other personnel will think that you are really mad [mentally ill], since you are working with them” (Provider 1, Clinic 2).* Mental health-related stigma affected providers and clients, who both experienced concern that others would believe they had mental disorders based on their physical proximity to or collaboration with specialized mental health providers.

Providers also described that limited community awareness about mental disorders and treatment was a barrier to clients’ mental health-related care seeking. Providers explained that people in Cameroon often “*don’t consider anxiety or depression to be a disease that can make you insane” (Provider 5, Clinic 2)*, therefore, it took time for clients to accept care. One provider elaborated on how limited community awareness may have been a barrier to clients accessing needed services:

*“The first thing for the patient is to accept they have a problem and come to the hospital to ask about their problem. Because the majority of people with related problems, with mental issues, don’t even accept the fact that they have mental problems. Generally, even in their circle, people around them do not accept them. They always try to associate it with a mystical issue, a question of bad luck. So, they prefer to go to the* tradipraticien *[traditional doctor] and so forth” (Provider 2, Clinic 2)*.

Referent individuals – here discussed as “in their circle, people around them” – may have influenced clients’ decisions to seek care from formal or traditional sources. This provider went on to say that due to limited community awareness and low levels of formal help-seeking, mental health “*remains a very serious problem in Cameroon.” (Provider 2, Clinic 2).*

Providers suggested that addressing mental health-related stigma and increasing community awareness through community education could improve their ability to effectively screen clients for mental disorders. Providers described these efforts as *“sensitization”*, *“awareness campaigns”*, or even *“short videos or documentaries on mental health.”* One provider suggested that such educational campaigns could be integrated into the HIV facility in the form of daily morning health talks, citing the success of previous health talks about monitoring one’s blood pressure (BP):

*“For example, let me call this idea of taking vital signs. Especially the BP, when they started with it, so many of them they start complaining, oh they want to go back home early. But when it was being put on health talk – the importance of taking your BP – most of them they even come saying that, ‘hey doctor, let’s do my BP. Check my blood. Let’s see how I did’” (Provider 1, Clinic 1).*

This provider implied mental health-focused health talks could have similar success as the BP-focused talks and may enhance receptivity to subsequent mental health screening and services.

Providers believed they would also benefit directly from education initiatives, specifically mental health training. Providers suggested activities that included both mental health-related stigma reduction and mental health training and education could improve providers’ interactions with clients, facilitate help-seeking if they were experiencing mental health challenges themselves, and reduce mental health-related stigma within the facility. One provider discussed the success of an initiative to raise awareness among facility staff to address the stigma associated with the mental health unit:

*“Well, it is known, there was this prejudice attached to the mental health unit and the care provided in that unit. In the perception of even some personnel—and there are many [of them]—the mental health unit was directly linked to mental illness. […] When we were talking about the mental health unit, we were thinking more about—excuse the expression—“mad people”, in quotes. And then, as we started to work on raising awareness among the staff to explain to them what our service is, our unit and the care we offer, these prejudices have dissipated a little bit” (Provider 4, Clinic 3)*.

Providers discussed how limited mental health training influenced their beliefs and clinical care. Providers noted that additional mental health training could improve service quality by helping providers *“consider them [clients] as full-fledged men, with the same intelligence and everything that goes with it” (Provider 4, Clinic 3)*.

### Physical space and clinic volume

Nearly all providers (12/14) cited limited physical space as a barrier to integrating mental health screening at their facilities because it impacted confidentiality and client openness. Without adequate space, it was difficult to have sensitive conversations with clients who may not have felt comfortable discussing their mental health. One provider explained, *“if we have enough space to screen them privately, they’ll be able to maybe explain more of their problems” (Provider 1, Clinic 3).* Providers commented on the importance of clients’ demeanor during screening. Client demeanor conducive for screening was described as *“when your patient is open to you, when the patient is lively” (Provider 2, Clinic 1)*. Limited physical space was exacerbated by high clinic volume, which further hampered client openness. High clinic volume also contributed to long wait times and led to clients feeling frustrated and impatient during clinical visits. One provider mentioned they found it challenging to screen for mental health issues when clients were irritated from having to wait so long, saying *“by the time you talk, you feel like they have problems when they don’t have problems” (Provider 2, Clinic 2)*. Providers were cognizant of their clients’ frustration with long wait times and reported when their clients appeared to be in a hurry or pressed for time, providers felt they just “*don’t really have that time to start screening” (Provider 2, Clinic 1)*.

High clinic volume also impacted providers’ time and availability. One provider described that with limited time, the only way they would become aware of their clients’ mental health challenges is if the clients volunteered that information themselves: *“Each day we can see about 400 to 200 clients, 200 to 400 clients a day. And so it is impossible to identify who is suffering. Except they come out with their complaints” (Provider 3, Clinic 1)*. Other providers remarked that providing mental health care beyond screening took time, and with the number of clients they needed to see daily they were unable to incorporate counseling into their client interactions.

Providers at all facilities recommended provision of “more health staff” trained to conduct mental health screening to address high provider workloads or staff turnover or reassignment. Providers often recommended mental health personnel be integrated into the HIV treatment facility or to *“create a mental health department closely linked with the HIV unit” (Provider 2, Clinic 3)*, especially considering how frequently mental health challenges can occur among PWH. While providers most often attributed lack of client openness to limited physical space or privacy for mental health screening, they also discussed how provider demeanor, or their *“manner of approach”* was a key factor in fostering clients’ comfort. Providers believed nurturing positive relationships would help clients feel more open to disclosing mental health challenges they may experience.

While a provider’s poor demeanor could be attributed to environmental challenges at the facility (e.g., limited time, high client volume), mental health-related stigma was also noted as a barrier to establishing good relationships with clients. Training was cited as a mechanism to address mental health-related stigma and facilitate earlier detection of mental health problems. Further, providers explained that provider training could also help improve client demeanor if it reduced the need to refer clients for mental health care.

### Managing referrals

Coordination and communication presented a barrier to referring clients to mental health care within and outside the HIV treatment facilities. One provider cited *“patient flow”* as the biggest challenge at their facility, explaining PWH were unsure where else in the facility to access non-HIV specific services and that the distance between the HIV and mental health units is “*too far*.” Another provider at the same facility suggested that by *“putting in place a patient flow that would shorten the waiting time for the patients they would be more open and give us reliable and more coherent information, which would allow us to make a diagnosis” (Provider 2, Clinic 2)*. Two providers at different facilities alluded to the need for coordination across the entire facility by indicating even those at reception needed to be able to recognize signs and symptoms of mental health issues to facilitate referrals to the appropriate setting.

Despite several providers mentioning client referral, not all providers were aware of to whom to refer clients and rarely followed up to confirm whether referred clients were successfully linked to mental health care. One provider said, *“I don’t know specifically who was trained but I was told that some people were trained on this mental health problem” (Provider 4, Clinic 1)*, indicating that providers may have been unable to refer clients because they were unsure of appropriate services. One provider noted, *“we don’t actually try to follow up like the review from the mental health personnel to see if the patient actually met with them and what were their discussions” (Provider 5, Clinic 3)*. Providers also described stigma as a factor that contributed to clients not successfully linking to mental health referrals, with one provider explaining *“when we send them to psychiatry after diagnosing these problems they don’t want to go there” (Provider 5, Clinic 2)*. Providers noted that the lack of provider follow-up had repercussions on their client’s health, only learning a client was not successfully linked when they see the client *“at a very later stage for conditions that would be handled early” (Provider 3, Clinic 3)*, and that *“at the end of the day, [if the] patient no see the psychiatrist, he [goes] back with the same problem” (Provider 1, Clinic 2)*. Providers indicated that a stronger mental health referral process could improve patient health and wellness.

### Provider motivation and confidence

Despite the barriers previously noted, providers across participating clinics reported being motivated to integrate mental health screening into HIV care. They saw their role as helping clients holistically with their health and recognized mental health as part of providing the best care possible to clients: *“We are health professionals, so our motivation is to do everything we can for the well-being of all patients. So, in that respect, that’s the motivation. We need to make sure that any patient who has a problem can be in good health again” (Provider 2, Clinic 2)*. In addition, providers described their motivation to integrate mental health screening and services because they believed it would result in furthering HIV-related health goals such as reducing clients’ viral load:

*“What would encourage us is that, if these patients who have mental problems are mentally stable, it is a big gain for us because it will allow us to maintain them or to achieve viral suppression […] Because, knowing that – if they have a mental pathology that is not taken care of, it is also a factor that lowers their immunity. And even if they’re taking their ARV [antiretroviral medication] correctly, just that one factor—if it’s not taken care of—can destabilize everything. So, it [mental health screening and treatment] would allow us to take better care of our patients” (Provider 5, Clinic 2)*.

All providers said they would feel confident screening for mental disorders with adequate training and resources. One provider described the relationship between training and confidence by saying, “i*f you are trained, you will know exactly what to do so you will have the confidence” (Provider 1, Clinic 3)*. Some providers described that their confidence stemmed from the good rapport they already established with their clients: “*Because I have been with the patients for long, I have been in this center for more than seven years, so I know how to deal with them” (Provider 2, Clinic 1).* Providers shared their understanding of mental health screening as a systematic process that could lead to being able to *“identify more and more patients with mental problems in a very reliable way” (Provider 2, Clinic 2)* and that with adequate training “*screening will be, in any case, more efficient. There are cases that are not – that pass [right] under our noses that we will now detect” (Provider 5, Clinic 2).* One provider – who shared they were trained in mental health care – elaborated that training needs to be ongoing and sustainable to be successful:

*“The first challenge is to organize ongoing training to update knowledge, to allow all the health care providers of this day care [HIV treatment center] service to have the same level of knowledge concerning mental health problems. […] We need to update our knowledge constantly to make sure that we are always up to date” (Provider 4, Clinic 3)*.

Several providers discussed how ongoing supervision would contribute to provider accountability and professional growth. One elaborated on the need for system-level accountability and leadership buy-in:

“*When I talk of ‘reliable system,’ let it not be like they came and launched training. Ask us to do this. All of a sudden, then you don’t hear from them, they are no more serious. You yourself, it will not motivate you. I would like a system that is continuous functioning and communication will motivate me. […] Yeah. They should make sure I send a report of what is happening to follow me more strictly” (Provider 1, Clinic 2)*.

This concept of a “reliable system” was mentioned frequently by participants in the context of provider motivation, accountability, and the need for leadership buy-in for mental health screening initiatives to be sustainable. Limited accountability at the leadership level – limited supervision or attention to sustainability when initiating a program – impacted provider motivation. While many levels within the health facility were involved in the success of screening and treatment of mental disorders due to needed collaboration across different units, one provider underscored the Ministry of Health’s responsibility to offer support as well. For any mental health screening initiatives to be successful, providers discussed the need for leadership buy-in at all levels and processes in place to support personnel accountability and program sustainability.

## Discussion

This research explored factors influencing the integration of mental health screening and treatment into HIV care from the perspective of health providers working at three HIV treatment centers in Cameroon. Providers discussed several barriers to integrate screening PWH for mental disorders, including lack of a standardized screening process, mental health-related stigma, limited community awareness about mental disorders, limited physical space, high clinic volume, and inadequate coordination to manage referrals. To support sustainable integration of mental health screening and treatment into HIV clinics in Cameroon, providers recommended community sensitization, training and hiring more staff to conduct mental health screening, improved coordination to manage referrals, and leadership buy-in at multiple levels of the health system.

To address stigma and limited community awareness about mental disorders, providers recommended community sensitization and stigma reduction activities. While mass media campaigns are a potential large-scale approach, evidence on their effectiveness to reduce mental health stigma is equivocal in high-income countries and the cost of implementing these campaigns is likely prohibitive in resource-limited settings [34]. Further, while there may be a relationship between knowledge and stigma, education initiatives alone may be insufficient to achieve meaningful mental disorder-related stigma reduction at the community level. For example, a 3-day school-based educational intervention in Nigeria found an increase in mental health-related knowledge among school youth but no effect on attitudes or desire for social distance from people with mental disorders [35]. More research is needed to determine the type of community sensitization efforts and the amount and type of exposure that could translate to mental health stigma reduction. Facility-based awareness-raising efforts –such as the health talks recommended by providers in this research –may be a feasible alternative to broader mass media or community-based awareness-raising interventions in Cameroon. Future research should examine the extent to which facility-based health education activities focused on mental health are associated with increased knowledge and acceptability of mental health screening and reductions in mental health-related stigma.

Leveraging informal sources of care may be an opportunity to address the stigma-related barriers to formal help seeking. One provider mentioned clients sometimes associate depression with “mystical issues” or “bad luck” and therefore may prefer to visit traditional healers. Witchcraft or spirits have been cited in some communities in sub-Saharan Africa as causal attributions for mental disorders [6, 36], and it has been estimated that half of people seeking mental health care in sub-Saharan Africa choose to first visit a traditional or religious healer [37]. Qualitative research suggests that some people may prefer visiting traditional or religious healers when experiencing psychological distress and may have better access to these sources compared to formal medical providers, especially in low resource settings [38]. An integrated mental health and HIV pilot intervention in Zimbabwe involving nurses, community health workers, and traditional healers revealed all provider types found the intervention feasible and acceptable, with “traditional medicine practitioners highly valu[ing] their involvement, as it allowed for a clear and recognized pathway to collaborate with the formal health system, further validating their role with the Ministry of Health” [39]. Partnering with traditional or faith healers may be an opportunity to improve access to mental health care for PWH in Cameroon. Future programs should consider participatory approaches to intervention development and adaptation, which have been shown to contribute to community buy-in and local ownership and ensure that programs are culturally appropriate [40].

Providers advocated for training to reduce provider mental health-related stigma and increase their confidence to screen their clients for mental disorders. Providers reported their colleagues sometimes expressed concerns that working with clients with mental disorders might lead to assumptions about their own mental health status, consistent with qualitative research in Guinea finding medical students’ mental health-related stigma reflected that of the general population [36]. Perceived provider stigma has been found to impact clients’ internalized stigma and feelings of disempowerment [41]. Training interventions to address mental health-related stigma among primary care providers and community health workers in sub-Saharan Africa have improved mental health knowledge and attitudes towards people with mental disorders [34, 42, 43]. However, stigma reduction research has demonstrated that contact with people with mental disorders is more effective than interventions with only educational components, and that multiple encounters are more likely to be successful than one-time contact [44]. The RESHAPE program (Reducing Stigma among HealthcAre Providers) piloted in Nepal 2016–2018 supplemented standard World Health Organization Mental Health Gap Action Program (mhGAP) training with testimonials from PWH with lived experience of mental disorders. RESHAPE participants exhibited greater decreases in stigmatizing attitudes, improved diagnostic accuracy, and increased willingness to initiate psychological services compared to providers receiving standard mhGAP training [45, 46]. Adequately training staff to successfully integrate mental health care may also reduce mental health-related stigma. A qualitative study in Guinea found that providers in facilities that integrated mental health care had more positive attitudes towards people with mental disorders compared to providers in facilities where mental health care was not integrated [47]. This success was attributed to the training and improved confidence after successfully treating clients [47].

Currently, there is an opportunity to augment providers’ formal mental health training in Cameroon. In 2015 Cameroon incorporated a mental health curriculum into medical school training, and recently opened a psychiatric nursing school, though it is unclear the degree to which mental health is incorporated into nurses’ general education [24]. Relatedly, a 2018 cross-sectional analysis in Cameroon’s Fako Division found that 84% of general practitioners and 47% of nurses reported receiving any training on mental disorders [25]. Decisions to offer additional capacity-building opportunities to providers should also consider provider availability. Despite high provider-reported motivation, providers reported limited time to conduct screening, especially if they worked in a high-volume facility. High workload has been shown to negatively impact intervention effectiveness, and accommodating providers’ schedules to organize multiple trainings might pose implementation challenges [48, 49]. Implementing interventions in groups or providing ongoing supportive supervision for on-the-job training may address some of the limitations with provider availability, anchor training concepts in practice, and ultimately contribute to longer term intervention sustainability [17, 48, 49].

Providers recommended hiring more staff to conduct mental health screening and treatment to address human resource challenges. While it is likely not feasible to integrate specialized mental health providers into all HIV treatment facilities given human resources constraints, task-sharing or task-shifting (i.e., training non-specialized personnel to detect, screen, and manage mental disorders) has been shown to be feasible in sub-Saharan African settings, including Cameroon [50, 51]. Considering high provider motivation to conduct mental health screening, task shifting is a potential approach to address mental health needs of PWH in these HIV treatment facilities. A task-shifting intervention that trained non-psychiatric providers to prescribe and manage medical treatment for depression piloted in Bamenda, Cameroon was found to be feasible, acceptable, safe, and demonstrated preliminary efficacy in improving depression symptoms and HIV clinical outcomes [51, 52]. Evidence-based tools such as the mhGAP already exist for non-specialists to deliver mental health care in primary care and community-based settings [53]. Systematic reviews of evidence on mhGAP implementation have demonstrated that learners have improved knowledge, attitudes, and confidence after participating in mhGAP training [54, 55]. However, it is important to consider sociocultural context when adapting global standardized instruments such as mhGAP to new contexts [55, 56]. Additionally, long, complex tools covering multiple mental disorders such as the mhGAP can place additional burden on providers and clients alike, limiting widespread adoption. Brief, efficient, evidence-based tools are needed in order for task-shifting of mental health screening to be cost effective and time efficient in low-resource settings. For example, the mental wellness tool (mwTool) screens for eight mental disorders with 12 items and has demonstrated high sensitivity and specificity [57].

To ensure clients are successfully referred, providers recommended improving coordination and communication within and between facilities. Providers shared they weren’t always aware of the client referral processes, and providers rarely followed up to ensure clients were successfully linked to referrals. Limited knowledge on appropriate referral pathways is unsurprising given the limited standard mental health screening processes or tools. Guidelines that delineate appropriate referral pathways may improve providers’ capacity to screen for mental disorders. In Cameroon’s Fako division, 21.6% of public facility-based health providers said they would send their clients home or to a traditional healer if their client was experiencing depression [25]. In Zimbabwe, a pilot intervention that trained community health workers and traditional healers on a brief mental health screening tool to implement during routine HIV services resulted in 83–93% of those screened positive being referred to a health facility; however, in some instances nurses did not perceive the referrals from community health workers or traditional healers as legitimate [39]. While interventions to improve community-facility referral pathways may improve bidirectional referrals, it is important that providers trust each other. In addition, referral linkages that include follow-up may improve the likelihood that clients will successfully be engaged in mental health care. Studies examining referral processes for community- or home-based HIV testing find that facility linkage and ART initiation was higher in interventions with versus without facilitated linkage to a health care facility (e.g., someone designated to follow up and support people newly diagnosed getting connected to healthcare) [58].

Providers emphasized the need for leadership buy-in and accountability at multiple levels of the health system for mental health integration in HIV care to be sustainable. Providers shared that leaders who do not emphasize accountability or sustainability can hamper their motivation to screen for mental disorders. One third of countries globally do not have any budget earmarked for mental health, and most countries in Africa dedicate < 1% of their health budgets to mental health [15]. Developing and disseminating evidence-based processes for mental health screening, management, and referral and allocating sufficient resources to support system-wide coordination of mental health care demonstrates political will. Resource allocation should reflect efforts to decentralize services and ensure that infrastructure needs are being met. Leaders’ decisions to upgrade physical infrastructure should consider providers’ reports that physical space constraints impeded their ability to conduct confidential mental health screening, a similar finding to other qualitative research investigating health system preparedness for mental health service integration [59]. Physical infrastructure upgrades could also prioritize a patient-centered approach and consider ways to improve patient flow and facilitate internal referrals. Supply chain management plans to ensure reliable and sustainable procurement and distribution of psychiatric medication are also warranted, as interventions incorporating pharmacological and psychological treatments may be more effective than either type of intervention independently [60].

### Limitations

This study has limitations worth noting. The first author (KG) joined this research project after data collection was completed. Recognizing her position as a US-based researcher, KG engaged in reflexive memo-writing to think critically, challenge assumptions, and recognize how her lived experience shaped her interpretation of the data [61]. To ensure that themes and interpretations presented in this article uphold participants’ experience, coauthors with extensive familiarity with Cameroonian HIV clinical context and engagement during data collection checked interpretations. In addition, while our findings present important insights for integrating mental health care in HIV treatment facilities throughout Cameroon, participating facilities were all located in urban areas. Previous research has highlighted substantial gaps for mental health management in rural compared to urban settings [12]. As such, these findings should be interpreted accordingly. Lastly, to protect participant anonymity, we did not collect sociodemographic information from providers and are unable to report on whether providers’ experiences varied by such characteristics or role within the facility.

## Conclusion

Integrating mental health care into HIV treatment centers is a strategy to address unmet mental health needs among PWH in Cameroon. Providers reported enthusiasm to integrate mental health services into HIV care but needed more support and training to do so in an effective and sustainable manner. Developing and disseminating evidence-based processes for mental health screening, management, and referral; allocating sufficient resources to support system-wide coordination of mental health care; and conducting training and supportive supervision may result in improved provider confidence, motivation, and capacity to screen and treat PWH for mental disorders in Cameroon.

### Supplementary Information

**Supplementary Material 1.**

## Data Availability

Upon request, data are available to interested parties pending IRB approval from the University of North Carolina and the National Ethics Committee of Cameroon. Requests for the data can be sent to irb_questions@unc.edu.
