# Supplementary material for: Factors influencing integration of mental health screening and treatment at HIV clinic settings in Cameroon: a qualitative study of health providers’ perspectives
Source: BMC Health Serv Res. 2024 Apr 24;24:519. doi: 10.1186/s12913-024-10775-w (PMC11044447; doi:10.1186/s12913-024-10775-w)
Supplement: Supplementary file 1 — Supplementary Material 1. [file 12913_2024_10775_MOESM1_ESM.docx]

**In-depth interview guide – staff participants**

The purpose of this interview is to help doctors and healthcare providers understand how best to integrate mental health screening and treatment into HIV care. Your opinion is really important to figure out what things might make integrating mental health screening and treatment easier for people living with HIV. Remember that you never have to answer any question if it makes you uncomfortable.

1. What do you think are the biggest mental health challenges of patients at this HIV clinic?
2. What is the current process for screening for mental health problems?
3. To what extent does the physical environment facilitate or hinder you from screening for mental health problems?

- Prompt to consider: physical space to screen patients, competing tasks or time constraints, knowledge or training

1. To what extent do social influences facilitate or hinder you from screening for mental health problems?

Prompt to consider: Other providers screening for mental health, support or supervision for screening, availability of screening protocols

1. What challenges do you think there would be to screen and manage mental health problems at this clinic?
2. What support would you need to screen and manage mental health problems?
3. How confident are you that you could screen for mental health problems with appropriate training and supervision?
4. What would motivate you to screen for mental health problems?
5. What steps would this health facility have to take to help you screen for mental health problems?
6. Is there something that we didn’t talk about that want to share? Are there any questions you have for the researcher?

Thank you very much for your time and responses.
